# Diagnostic Efficiency of Three Fully Automated Serology Assays and Their Correlation with a Novel Surrogate Virus Neutralization Test in Symptomatic and Asymptomatic SARS-COV-2 Individuals

**DOI:** 10.3390/microorganisms9020245

**Published:** 2021-01-25

**Authors:** Salma Younes, Hadeel Al-Jighefee, Farah Shurrab, Duaa W. Al-Sadeq, Nadin Younes, Soha R. Dargham, Nader Al-Dewik, Hamda Qotba, Mohamed Syed, Ahmed Alnuaimi, Hadi M. Yassine, Patrick Tang, Laith J. Abu-Raddad, Gheyath K. Nasrallah

**Affiliations:** 1 Biomedical Research Center, Qatar University, Doha P.O. Box 2713, Qatar; salma.nagy.younes@gmail.com (S.Y.); hadeel.mohammed@qu.edu.qa (H.A.-J.); farah.shurrab@qu.edu.qa (F.S.); dalsadeq@qu.edu.qa (D.W.A.-S.); nyounes@qu.edu.qa (N.Y.); hyassine@qu.edu.qa (H.M.Y.); 2 Department of Biomedical Science, College of Health Sciences, Member of QU Health, Qatar University, Doha P.O. Box 2713, Qatar; 3 College of Medicine, Member of QU Health, Qatar University, Doha P.O. Box 2713, Qatar; 4 Infectious Disease Epidemiology Group, Weill Cornell Medicine-Qatar, Cornell University, Qatar Foundation–Education City, Doha P.O. Box 24144, Qatar; srd2003@qatar-med.cornell.edu (S.R.D.); lja2002@qatar-med.cornell.edu (L.J.A.-R.); 5 World Health Organization Collaborating Centre for Disease Epidemiology Analytics on HIV/AIDS, Sexually Transmitted Infections, Viral Hepatitis, Weill Cornell Medicine–Qatar, Cornell University, Qatar Foundation–Education City, Doha, P.O. Box 24144, Qatar; 6 Clinical and Metabolic Genetics Section, Pediatrics Department, Hamad General Hospital (HGH), Hamad Medical Corporation, Doha P.O. Box 3050, Qatar; naldewik@hamad.qa; 7 Qatar Medical Genetic Center and Interim Translational Research Institute, Hamad Medical Corporation, Doha P.O. Box 3050, Qatar; 8 College of Health and Life Science, Hamad Bin Khalifa University, Doha P.O. Box 34110, Qatar; 9 Department of Pediatrics, Women’s Wellness and Research Center, Hamad Medical Corporation, Doha P.O. Box 3050, Qatar; 10 Department of Clinical Research, Primary Health Care Centers, Doha P.O. Box 26555, Qatar; haqotba@phcc.gov.qa (H.Q.); masyed@phcc.gov.qa (M.S.); asalnuaimi@phcc.gov.qa (A.A.); 11 Department of Pathology, Sidra Medicine, Doha P.O. Box 26999, Qatar; ptang@sidra.org; 12 World Health Organization Collaborating Centre for Disease Epidemiology Analytics on HIV/AIDS, Sexually Transmitted Infections, and Viral Hepatitis, Weill Cornell Medicine–Qatar, Cornell University, Qatar Foundation–Education City, Doha P.O. Box 24144, Qatar; 13 Department of Healthcare Policy and Research, Weill Cornell Medicine, Cornell University, New York, NY 10021, USA

**Keywords:** COVID-19, SARS-CoV-2, serology, sensitivity, specificity, neutralizing antibodies, surrogate virus neutralization test (sVNT)

## Abstract

To support the deployment of serology assays for population screening during the COVID-19 pandemic, we compared the performance of three fully automated SARS-CoV-2 IgG assays: Mindray CL-900i^®^ (target: spike [S] and nucleocapsid [N]), BioMérieux VIDAS^®^3 (target: receptor-binding domain [RBD]) and Diasorin LIAISON^®^XL (target: S1 and S2 subunits). A total of 111 SARS-CoV-2 RT-PCR- positive samples collected at ≥ 21 days post symptom onset, and 127 pre-pandemic control samples were included. Diagnostic performance was assessed in correlation to RT-PCR and a surrogate virus-neutralizing test (sVNT). Moreover, cross-reactivity with other viral antibodies was investigated. Compared to RT-PCR, LIAISON^®^XL showed the highest overall specificity (100%), followed by VIDAS^®^3 (98.4%) and CL-900i^®^ (95.3%). The highest sensitivity was demonstrated by CL-900i^®^ (90.1%), followed by VIDAS^®^3 (88.3%) and LIAISON^®^XL (85.6%). The sensitivity of all assays was higher in symptomatic patients (91.1–98.2%) compared to asymptomatic patients (78.4–80.4%). In correlation to sVNT, all assays showed excellent sensitivities (92.2–96.1%). In addition, VIDAS^®^3 demonstrated the best correlation (r = 0.75) with the sVNT. The present study provides insights on the performance of three fully automated assays, which could help diagnostic laboratories in the choice of a particular assay according to the intended use.

## 1. Introduction

The COVID-19 pandemic, caused by the severe acute respiratory syndrome coronavirus 2 (SARS-CoV-2) [1,2] was first reported in December 2019 in Wuhan, China [3,4]. The virus has rapidly spread and become a major public health concern, resulting in a total of 80,818,467 confirmed cases and 1,766,847 deaths, as of 27 December 2020 [5].

Although molecular detection techniques have played an important role in testing and contact tracing efforts, virus elimination is perhaps no longer feasible due to the extensive and insidious spread of the virus. Thus, further diagnostic methods are needed to guide the most efficient use of public health measures. The gradual lifting of restrictions and control measures will require active surveillance to allow early detection of new cases or clusters, along with retrospective contact tracing and quarantine, most likely combined with physical distancing measures and augmented protection of those at higher risk. Serology testing is ideally suited for this purpose as it can inform the need for contact tracing, investigation of asymptomatic and other undocumented infections, accurate determination of the infection fatality rate, assessment of herd immunity, and the level and duration of protective immunity in the population at large and in specific groups [6], which remains a key knowledge gap in COVID-19 research.

Laboratories and companies are racing to produce reliable and versatile serological tests that can detect SARS-CoV-2 infection with sufficient specificity and sensitivity [6]. The required performance of a serological test will depend on the purpose of testing. Numerous commercial serological tests have been developed and introduced into the market [7,8]. However, due to the need for their rapid development and implementation, in many countries, the normally stringent regulatory criteria have not been applied to many of them [6]. Thus, persistent concerns remain regarding the accuracy and reliability of the currently available SARS-CoV-2 immunoassays.

Serological tests typically detect antibodies against spike protein (S) and/or nucleoprotein (N) since these are the most immunogenic proteins of SARS-CoV- 2 [9]. Recently, it has been shown that antibodies directed against the S1 subunit of the SARS-CoV-2 S protein, specifically against the receptor-binding domain (RBD), strongly correlate with virus neutralization [9]. Thus, the likelihood of predicting protective antibody responses increases when using either the S1 antigens or the RBD in the assay. The specificity of antibody tests in detecting antibodies against SARS CoV-2 might be hampered by the presence of antibodies against other circulating coronaviruses in the population [10], and thus, testing for cross-reactivity is crucial. When selecting an appropriate antibody test for a specific aim, it is necessary to develop a broad understanding of antibody specificities, kinetics, and functions [11]. The lack of knowledge of antibody kinetics in emerging viral infections during an outbreak is always a challenge for validation of serological tests. Recent studies on have shown that seroconversion rates have reached as high as 100% after 10–14 days, and that antibody levels correlate with clinical severity [9,12,13]. This is in concordance with reports on the Middle East Respiratory Syndrome coronavirus (MERS-CoV) infection, in which antibody response varies according to disease severity, with mild and asymptomatic infections resulting in weaker immune responses [12]. Thus, sufficient samples from persons with mild and asymptomatic disease should be included in validation studies for useful interpretation and extrapolation of results to population screening.

In the present study, we aimed to evaluate the performance of three commercially available automated analyzers for the detection of anti-SARS-CoV-2 IgG antibodies using confirmed RT-PCR samples that were collected from symptomatic and asymptomatic RT-PCR confirmed cases. In addition, for the first time, we assessed the performance of the three commercial automated assays in correlation to a surrogate virus neutralization test (sVNT). The CL-900i^®^ detects anti-S and anti-N antibodies, LIAISON^®^XL-Diasorin detects anti-S1 and anti-S2 antibodies, and VIDAS^®^3- bioMérieux detects antibodies directed against the RBD of the S1 subunit. We assessed the sensitivity, specificity, Cohen’s Kappa, and estimated the positive and negative agreement values of the three automated assays in correlation to the gold standard RT-PCR, and the sVNT. We also performed concordance assessment among the assays. The strength of this study lies in the diversity of the sample population characteristics, with ~89% of the total population of Qatar being expatriates from over 150 countries [13,14,15,16].

## 2. Materials and Methods

### 2.1. Study Design, Ethical Approval, and Clinical Samples

We evaluated the performance of three CE-marked fully automated analyzers: CL-900i^®^ (Mindray Bio-Medical Electronics Co., Shenzhen, China), VIDAS^®^3 (bioMérieux, Marcy-l’Étoile, France) and LIAISON^®^XL (DiaSorin, Saluggia, Italy) for detecting anti-SARS-CoV-2 IgG antibodies. This project was approved by Center for Disease Control (CDC) at Hamad Medical Corporation (HMC), the Primary Health Care Corporation (PHCC) and Qatar University (QU). 

All specimens used in the study were in a hospital setting, or professional laboratory acquisitioned for routine testing, and shipped on ice packs to our laboratory. According to CDC recommendations, all samples were stored in a refrigerator at 4 ± 2 °C for up to 72 h after collection if a delay in shipping or processing was expected. Samples were centrifuged at 2500 rpm for 10 min to facilitate plasma/cell phase separation. The resulting upper plasma layer was extracted, and tested fresh, or aliquoted to minimize future freeze-thaw cycles, and stored at –80 °C for later analyses. Frozen samples were thawed on ice before the analysis.

To determine the specificity of each automated analyzer and to investigate cross-reactivity, we used a well-defined panel of pre-pandemic plasma samples collected from blood donors before 2019 and used in previous studies [17,18,19,20,21,22,23,24,25]. The panel comprised of 127 plasma samples seropositive for (a) other human coronaviruses (*n* = 18), (b) non-CoV respiratory viruses (*n* = 38), (c) non-respiratory viruses (*n* = 65), and (d) antinuclear antibodies (ANAs) (*n* = 6).

Sensitivity was determined using sera collected from 111 RT-PCR-confirmed SARS-CoV-2 patients, with different COVID-19 clinical outcomes. Qiagen RNA extraction kit was used to extract RNA from nasopharyngeal swab specimens. The extracted RNA was tested for SARS-CoV-2 using the SuperscriptIII OneStep RT-PCR kit (Cat No. 12594100, ThermoFisher, Waltham, MA, USA). Each sample was tested using three sets of primers: one set targeting the E gene for screening and the other two sets targeting the RdRp gene for confirmation as described in [26]. Cycle threshold (CT) values below 32 were considered positive. All samples were collected ≥21 days of symptoms onset. Clinical records of the patients were reviewed to determine the disease’s severity and were categorized into: (a) symptomatic (*n* = 56), and (b) asymptomatic (*n* = 51). All specimens were stored at −80 °C until use.

### 2.2. Automated-IgG Assays

Commercial automated analyzers from three different companies were used for the detection of anti-SARS-CoV-2 IgG antibodies in the sera of COVID-19 patients and the control group. These assays are: (i) CL-900i^®^ SARS-CoV-2 IgG (Cat. No. SARS-CoV-2 IgG121, Mindray, Shenzhen, China) [27,28] (ii) VIDAS^®^3 SARS-CoV-2 IgG (Cat. No. 423834, bioMérieux, Marcy-l’Étoile, France) [29,30], (iii) LIAISON^®^XL SARS-CoV-2 IgG (Cat. No. 311450, Diasorin, Saluggia, Italy) [30,31]. All tests were carried out according to the manufacturers’ instructions. The characteristics of the assays, including detection method, targeted antigens, sample volume, result interpretation, and reported sensitivity and specificity are summarized in Table 1.

### 2.3. Neutralization Assay (sVNT)

The SARS-CoV-2 surrogate virus neutralization test (sVNT) was used as a reference in this study (Cat. No. L00847, GenScript, NJ, USA) [32,33] for detecting neutralizing antibodies. This assay was developed by GenScript^®^ Biotech and is now available commercially as 96-well microplates for large serological screening for neutralizing antibodies targeting the RBD domain of the S1 subunit. Moreover, this assay demonstrated a high correlation with the pseudovirus neutralization test (pVNT, *R^2^* = 0.84) and the complete virus-neutralization test (cVNT, *R^2^* = 0.85) [33]. Validation of sVNT showed a specificity of 99.9% and a sensitivity of 95.0–100% [33]. In this study, all SARS-CoV-2 RT-PCR- positive plasma samples were tested for neutralizing antibodies against the RBD protein using the sVNT. According to the manufacturer’s instructions, a value result ≥20% signal inhibition was considered positive (neutralizing antibodies were detected), and <20% signal inhibition was considered negative (neutralizing antibodies were not detected).

### 2.4. Statistical Analysis

The diagnostic assessment of the three automated analyzers with RT-PCR for SARS-CoV-2 resulted in three cross-tabulations for each COVID-19 patient group versus the control group. Using RT-PCR as the reference standard, overall percent agreement, sensitivity, specificity, and Cohen’s Kappa statistic were calculated to assess the performance of each assay. Informed by literature, borderline results were considered positive [3,34].

Receiving operating characteristic (ROC) curves were conducted to study the diagnostic performance of each assay. The area under the curve (AUC) was estimated. Statistically, the larger the AUC, the more the accurate a tool can be considered in its overall performance. An AUC of 0.9–1.0 is considered excellent, 0.8–0.9 very good, 0.7–0.8 good, 0.6–0.7 sufficient, 0.5–0.6 bad, and less than 0.5 considered not useful [35]. The cut-off values for optimal sensitivity and specificity were determined by calculating Youden’s index J (J = sensitivity + specificity − 1). The Youden index J represents the point on the curve in which the distance to diagonal line (line of equality) is maximum [36].

Using the GenScript sVNT as the reference standard, the sensitivity for each automated analyzer was also calculated. Concordance analysis between the three automated assays along with the sVNT were conducted and resulted in 20 test combinations. These concordance measures include overall, positive, and negative percent agreement, as well as Cohen’s Kappa statistic. The latter measure is a standard and robust metric that estimates the level of agreement (beyond chance) between two diagnostic tests. Ranging between 0 and 1, a Kappa value <0.40 denotes poor agreement, 0.40–0.59 denotes fair agreement, 0.60–0.74 denotes good agreement, and ≥0.75 denotes excellent agreement [37]. The significance level was indicated at 5%, and a 95% confidence interval (CI) was reported for each metric. Pearson correlation coefficient (r) was calculated. For absolute values of Pearson’s r, 0–0.19 is denoted as very weak, 0.2–0.39 as weak, 0.40–0.59 as moderate, 0.6–0.79 as strong and 0.8–1 as very strong correlation [38]. All calculations were performed using GraphPad Prism Version 8.2.1.

## 3. Results

### 3.1. Diagnostic Performance Using RT-PCR as a Reference Test

**Figure 1:**
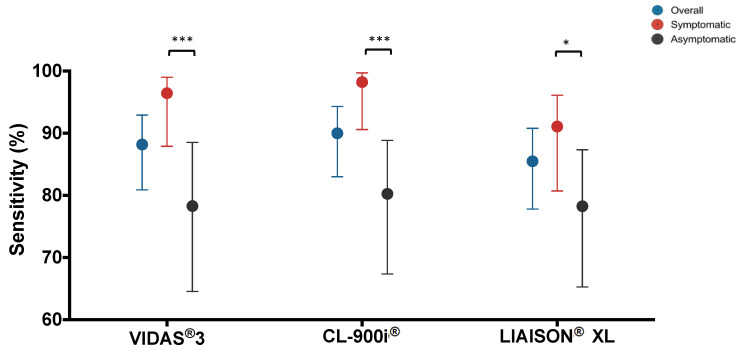
Sensitivity of each assay in samples collected ≥ 21 days post symptom onset using RT-PCR as a reference test. Data are presented for 111 RT-PCR confirmed SARS-CoV-2 samples categorized into: symptomatic (*n* = 56); asymptomatic (*n* = 51); and unclassified (*n* = 4); run on each automated assay; VIDAS^®^3, CL-900i^®^, LIAISON^®^XL. Chi-square test was used to detect the presence of a statistically significant difference in the sensitivity of each assay between the symptomatic and asymptomatic samples. * *p* < 0.05, *** *p* < 0.0001.

The overall diagnostic performance of each automated analyzer in comparison with RT-PCR is summarized in Table 2 and Figure 1. The overall percent agreement with RT-PCR was above 90% for all the three analyzers; VIDAS^®^3 93.7% (95% CI: 89.9–96.2%), CL-900i^®^ 92.9% (95% CI: 88.9–95.5%), and LIAISON^®^XL 93.3% (95% CI: 89.4–95.8%) (Table 2A). The highest sensitivity was estimated at 90.1% (95% CI: 83.1–94.4%) for CL-900i^®^ as shown in Figure 1. The highest specificity was estimated at 100% (95% CI: 97.1–100%) for LIAISON^®^XL (Table 2A). The Cohen’s Kappa statistic denoted excellent agreement for all three automated analyzers; VIDAS^®^3 at 0.87 (95% CI: 0.83–0.92); CL-900i^®^ at 0.86 (95% CI: 0.81–0.90); and LIAISON^®^XL at 0.86 (95% CI: 0.82–0.91) (Table 2A).

In symptomatic COVID-19 patients, the overall percent agreement with RT-PCR was above 95% for all the three analyzers; VIDAS^®^3 97.8% (95% CI: 94.5–99.1%); CL-900i^®^ 96.2% (95% CI: 92.3–98.1%), and LIAISON^®^XL 97.3% (95% CI: 93.8–98.8%) (Table 2B). The highest sensitivity was estimated at 98.2% (95% CI: 90.6–99.7%) for CL-900i^®^ as shown in Figure 1. The highest specificity was estimated at 100% (95% CI: 97.1–100%) for LIAISON^®^XL (Table 2B). The Cohen’s Kappa statistic denoted excellent agreement for all three automated analyzers; VIDAS^®^3 at 0.95 (95% CI: 0.20–0.98); CL-900i^®^ at 0.91 (95% CI: 0.87–0.95); and LIAISON^®^XL at 0.93 (95% CI: 0.90–0.97) (Table 2B).

In asymptomatic COVID-19 patients, the overall percent agreement with RT-PCR was above 90% for all the three analyzers; VIDAS^®^3 92.7% (95% CI: 89.8–96.2%); CL-900i^®^ 91.1% (95% CI: 85.9–94.4%), and LIAISON^®^XL 93.8% (95% CI: 89.3–96.5%) (Table 2C). The highest sensitivity was estimated at 80.4% (95% CI: 67.5–89.0%) for CL-900i^®^ as shown in Figure 1. The highest specificity was estimated at 100% (95% CI: 97.1–100%) for LIAISON^®^XL (Table 2C). The Cohen’s Kappa statistic denoted excellent agreement for all three automated analyzers; VIDAS^®^3 0.81 (95% CI: 0.75–0.87); CL-900i^®^ 0.77 (95% CI: 0.71–0.84); and LIAISON^®^XL 0.84 (95% CI: 0.78–0.90) (Table 2C). Furthermore, most tested performance parameters, particularly sensitivity, were greater in symptomatic samples than the asymptomatic (Figure 1).

**Figure 2:**
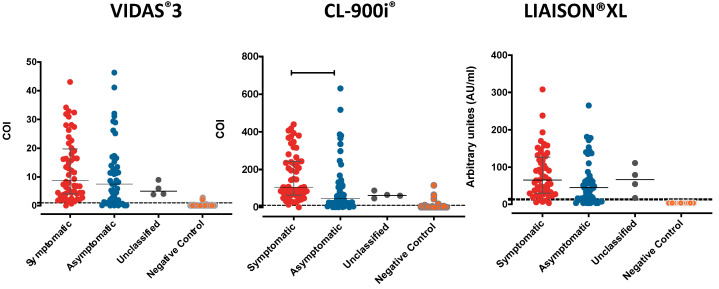
Distribution of numerical results obtained from each automated analyzer. Results are represented as dot plots to review the scatter of values around the prespecified assay cut-off (shown as dashed lines). Data are presented for 111 RT-PCR confirmed SARS-CoV-2 samples (symptomatic, asymptomatic, and unclassified), and 127 pre-pandemic control samples for each of the three automated assays. The dashed lines represent the cut-off values for the automated assays. The continuous lines represent the median and confidence interval (CI) for each group. One-way analysis of variance (ANOVA) was used to compare the differences between groups.

The overall distribution of the values generated by each automated analyzer against the cut-offs (dashed lines) is shown in Figure 2. As depicted in the figure, only CL-900i^®^ showed a significant difference between the symptomatic and asymptomatic samples (*p* = 0.0063), suggesting that CL-900i^®^ could be used in the future as a simi-quantitative assay by performing an in-point titration curve.

### 3.2. Evaluation of Potential Cross-Reactivity with Other Viruses

The specificity of each automated analyzer in relation to sample cross-reactivity with antibodies against various viruses is summarized in Table 3. Of the 127 pre-pandemic control samples, eight sera samples cross-reacted; two samples cross-reacted with VIDAS^®^3, while the remaining six samples cross-reacted with CL-900i^®^. In the other-coronaviruses subgroup, CL-900i^®^ demonstrated the lowest specificity at 66.7% (95% CI: 43.8–83.7%) compared to both VIDAS^®^3 and LIAISON^®^XL at 100% (95% CI: 82.4–100%). For non-CoV respiratory viruses (influenza A and RSV), both CL-900i^®^ and LIAISON^®^XL showed no cross-reactivity at a specificity of 100% (95% CI: 90.8–100%) for both; whereas VIDAS^®^3 cross-reacted with one sample demonstrating the lowest specificity among the three automated analyzers at 97.4% (95% CI: 86.4–99.5%). For non-respiratory viruses, no cross-reactivity was observed by the three automated analyzers, demonstrating a specificity of 100% (95% CI: 94.4–100%). For ANAs subgroup, both CL-900i^®^ and LIAISON^®^XL showed no cross-reactivity with a specificity of 100% (95% CI: 61.0–100%); whereas VIDAS^®^3 cross-reacted with one sample with a specificity of 83.3% (95% CI: 43.7–97.0%). 

### 3.3. Receiver Operating Characteristics (ROC) Curve Analysis

**Figure 3:**
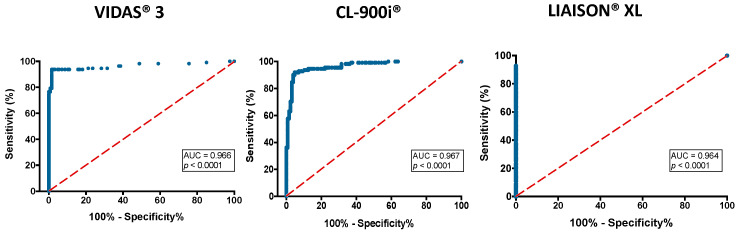
Receiver Operating Characteristic (ROC) curves for each automated analyzer. An AUC of 0.9–1.0 is considered excellent, 0.8–0.9 very good, 0.7–0.8 good, 0.6–0.7 sufficient, 0.5–0.6 bad, and less than 0.5 considered not useful [35].

ROC curve analysis was performed. As depicted in Figure 3, a performance with AUC >0.90, denoted as excellent performance was observed for all three automated analyzers; VIDAS^®^3: 0.97, CL-900i^®^: 0.97, LIAISON^®^XL: 0.96, with *p* < 0.0001. Based on the ROC curves, the optimized cut-off indices were derived. We chose optimal decision thresholds for cut-offs based on the Youden’s index (maximum sum of sensitivity and specificity). The cut-offs were >0.48, >6.83, and >4.78 for VIDAS^®^3, CL-900i^®^ and LIAISON^®^XL, respectively. The corresponding manufacturer’s suggested cut-offs were ≥1, >10, and 15, respectively. Applying these thresholds, the overall sensitivity of the VIDAS^®^3 assay improved (93.7% vs. 88.3%), and the specificity was unaffected (98.4%). CL-900i^®^ showed a slightly improved sensitivity (91.9% vs. 90.1%), while the specificity was unaffected (95.3%). Using the cut-off index for LIAISON^®^XL, the overall sensitivity improved significantly (92.8% vs. 85.6%), and the specificity remained at 100%.

### 3.4. Diagnostic Performance Using sVNT as the Reference Test

**Figure 4:**
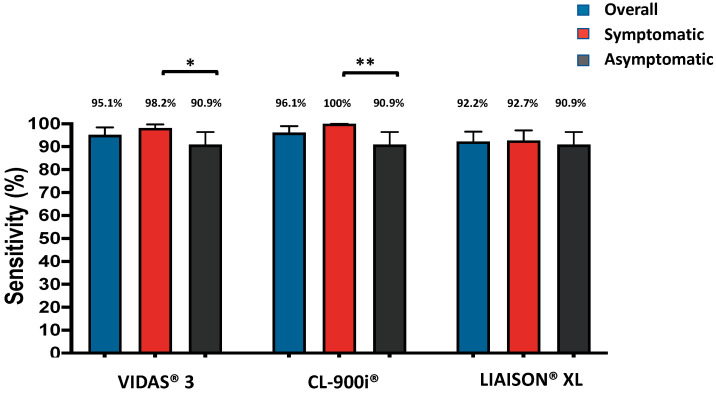
Sensitivity for each assay on samples collected ≥ 21 days post symptom onset in patients with SARS-CoV-2 RT-PCR-confirmed infection using the sVNT as a reference test. Data are presented for 111 RT-PCR confirmed SARS-CoV-2 positive samples categorized as: overall (*n* = 111), symptomatic (*n* = 56); and asymptomatic (*n* = 51); run on each automated assay; VIDAS^®^3, CL-900i^®^ SARS-CoV-2, and LIAISON^®^XL. Chi-square test was used to detect the presence of a statistically significant difference in the sensitivity of each assay between the symptomatic and asymptomatic samples. * *p* < 0.05, ** *p* < 0.001.

The sensitivities of the three automated analyzers, using GenScript sVNT as a reference assay, are summarized in Figure 4. As depicted in the figure, all three assays showed a sensitivity above 90%. The highest overall sensitivity was estimated at 96.1% (95% CI: 90.4–98.5%) for CL-900i^®^, followed by 95.1% (95% CI: 89.1–97.9%) for VIDAS^®^3 and 92.2% (95% CI: 85.4–96.0%) for LIAISON^®^XL. In symptomatic COVID-19 patients, the highest sensitivity was estimated at 100% (95% CI: 93.5–100%) for CL-900i^®^, followed by 98.2% (95% CI: 90.4–99.7%) for VIDAS^®^3 and 92.7% (95% CI: 82.7–97.1%) for LIAISON^®^XL. In asymptomatic COVID-19 patients, all three analyzers showed an equal performance, with a sensitivity of 90.9% (95% CI: 78.8–96.4%). 

### 3.5. Concordance Assessment among the SARS-CoV-2 IgG Automated Assays and the GenScript sVNT Test

The tests’ agreements were studied in a pairwise fashion applying inter-rater agreement statistics; (Cohen’s Kappa statistic, k) (Table 4). The overall percent agreement ranged from 92.8% (95% CI: 86.4–96.3%) for sVNT/LIAISON^®^XL test combination, and 95.5% (95% CI: 89.9–98.1%) for sVNT/VIDAS^®^3, sVNT/CL-900i^®^, and VIDAS^®^3/LIAISON^®^XL test combinations (Table 4). The positive percent agreement ranged from 92.2% (95% CI: 85.4–96.0%) for LIAISON^®^XL vs. sVNT to 100% (95% CI: 96.1–100%) and 100% (95% CI: 92.3–100%) for sVNT vs. LIAISON^®^XL and sVNT vs. VIDAS^®^3, respectively. The negative percent agreement ranged from 50.0% (95% CI: 28.0–72.0%) for sVNT vs. LIAISON^®^XL to 100% (95% CI: 67.6–100%) for VIDAS^®^3 vs. SVNT and LIAISON^®^XL vs. SVNT. Cohen’s Kappa statistic denoted good to excellent agreement and ranged between 0.63 (95% CI: 0.52–0.74); denoted as for LIAISON^®^XL/sVNT test combination and 0.80 (95% CI: 0.72–0.88) for LIAISON^®^XL/VIDAS^®^3 test combination. 

**Figure 5:**
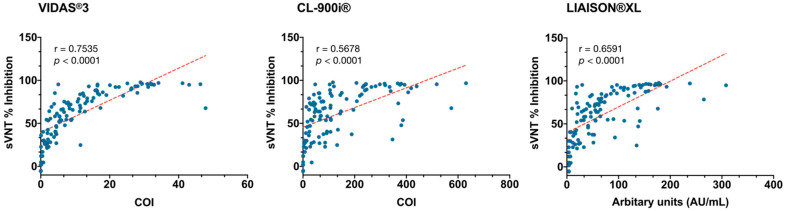
Pairwise correlational analysis of the assay numerical values obtained by each automated assay. Correlation plots of each automated assay with the sVNT. Pearson correlation coefficient (r) and *p*-value are indicated. Pearson’s r of 0–0.19 is regarded as very weak, 0.2–0.39 as weak, 0.40–0.59 as moderate, 0.6–0.79 as strong and 0.8–1 as very strong correlation, but these are rather arbitrary limits, and the context of the results should be considered. Data are presented for 111 RT-PCR confirmed SARS-CoV-2 positive samples and 127 known negative samples, run on each automated assay; VIDAS^®^3, CL-900i^®^ SARS-CoV-2, and LIAISON^®^XL.

A pairwise correlational analysis of the numerical values obtained by each automated IgG assay against the percentage inhibition obtained by sVNT was performed. As depicted in the correlation plots (Figure 5), all automated assays showed a moderate to strong correlation with the sVNT with Pearson’s r ranging from 0.5678 for CL-900i^®^/sVNT to 0.7535 for VIDAS^®^3/sVNT (Figure 5). Thus, VIDAS^®^3 demonstrated the best correlation with sVNT among all three automated IgG assays (Figure 5).

## 4. Discussion

The present study evaluated and compared the performance of three fully automated analyzers for the detection of SARS-CoV-2 IgG antibodies: CL-900i^®^, VIDAS^®^3, and LIAISON^®^XL. The sensitivity was evaluated using 111 samples collected from SARS-CoV-2 RT-PCR-positive symptomatic and asymptomatic patients. The specificity was evaluated using 127 pre-pandemic control samples. To assess the diagnostic performance of the three automated assays, RT-PCR was used as a reference test, in addition, for the first time, the performance of the three automated assays was assessed in correlation to a sVNT, which has recently been shown to correlate well with conventional virus neutralization test (cVNT), the current gold standard for the detection of neutralizing antibodies [39]. In this study, convalescent plasma samples (collected ≥21 days post symptom onset or positive PCR test) were used for the evaluation. It has been shown in several studies that most SARS-CoV-2 antibody assays exhibit variable performance during the early phases infection, but the concordance improves after day 14 of symptoms onset where IgG seroconversion rate reaches 90% [12,40,41]. According to recent data on COVID-19 serology testing, the performance of serological tests was found to stabilize ≥21 days after symptom onset [42]. Moreover, previous studies have shown that convalescent COVID-19 patients have higher neutralization activity [40,43]. Hence, these convalescent samples are expected to provide a more accurate evaluation of the selected assays. 

In the present study, we observed variable performance for the three automated assays. Among the three automated assays, CL-900i^®^ demonstrated the best overall performance in detecting SARS-CoV-2 IgG antibodies. The overall performance of the three assays was comparable to other detection methods such as Abbott Architect and Roche Cobas 6800, which were reported to have sensitivities of 93.5% and 95.2%, respectively, after 21 days of symptom onset, similar to CL-900i^®^ with showed the highest sensitivity (90.1%) (Table 2). Tang et al. reported a sensitivity of 89.4% by Roche assay [41], which was comparable to the sensitivity obtained by VIDAS^®^3 (88.3%). Another study on VIDAS^®^3 reported a sensitivity of 86.7% [30]; similar to our findings. Further, among the three automated assays, LIAISON^®^XL demonstrated the lowest sensitivity (85.6%) and failed to detect SARS-CoV-2 specific antibodies in three samples that were detected by both CL-900i^®^ and VIDAS^®^3. 

It is important to note that our COVID-19 cohort comprised both asymptomatic and symptomatic patients, of which most of the symptomatic cases were mild and non-hospitalized cases. Our study demonstrated that the sensitivity was higher in symptomatic patients compared to the asymptomatic patients (Table 2), which is in concordance with other studies reporting a stronger humoral immune response in severe COVID-19 patients compared to non-severe cases [43,44]. It is noteworthy to mention that among the 111 samples collected from SARS-CoV-2 RT-PCR-positive patients, nine were negative by all three assays, of which eight were collected from asymptomatic patients, and one was from a pauci-symptomatic patient. These patients may have developed a weak antibody response that was below the detection limit of the assays; thus, further investigation is needed by other highly sensitive assays. Also, a false positive PCR result or high CT-value (above 30 cycles) are plausible explanations, if an RT-PCR-positive COVID-19 participant had no detectable antibodies. It is noteworthy to mention that false positive PCR results due cross-contamination, or the interference of pure technical artifacts have been regularly documented even in the most highly regarded laboratories [45,46,47].

To assess the specificity of the automated assays, we have compiled pre-COVID-19 pandemic plasma sample obtained before the first appearance of the SARS-CoV-2 virus. Among the three assays, LIAISON^®^XL showed the highest specificity (100%), similar to a previous study from the United States that reported a 99.9% specificity [48]. However, the sensitivity of LIAISON^®^XL in our study using RT-PCR as the reference test was much lower (85.6%) compared to the one reported by the aforementioned study (100% by day 17 post symptoms onset) [48]. Overall, the specificity of all three analyzers was excellent, ranging from 95.3–100%). This is similar to what has been reported for other automated assys such as Abbot Architect™ i2000 (95.1%) and Elecsys^®^ Anti-SARS-CoV-2 (99.98%) reported elsewhere [35,49,50]. This could be due to the fact that both assays (Abbot Architect and Roche cobas™) are N protein-based which is conserved among coronaviruses leading to cross-reactivity. 

The variability in assay performance does not seem to be dependent on the different detection methods of each assay. CL-900i^®^, which is a CLIA-based assay, demonstrated the best performance compared to LIAISON^®^XL, which is also a CLIA-based assay that showed the lowest performance among all assays (Table 1 and Table 2 and Figure 1). However, this heterogenicity in assays performance is most likely dependent on the type of targeted antigen. The three automated assays were all based on different antigen components (Table 1). This is noteworthy, as antibody responses against each of these antigens may develop with variable kinetics, which remains a subject for further investigation. Our study showed higher specificities in assays targeting the S protein of SARS-CoV-2 (VIDAS^®^3 and LIAISON^®^XL) compared to the one targeting both S and N proteins (CL-900i^®^). This is because N protein is relatively small and more conserved than the S protein among human coronaviruses, which could cause false-positive results through cross-reactivity [51,52]. Therefore, although targeting both S and N proteins improved the sensitivity of CL-900i^®^, it decreased the specificity by causing cross-reaction with other coronaviruses. 

To determine which assay best correlate with neutralizing antibodies, GenScript sVNT test was used, a newly described VNT that has recently been shown to demonstrate an excellent performance in correlation to cVNT, the current gold standard for detecting neutralizing antibodies [33]. While cVNT provides the recognized benchmark, it is not practical for large-scale implementation due to requirement of a live pathogen, high biosecurity containment, and the need for highly trained personnel to perform the labor-intensive procedures. sVNT on the other hand, was designed to detect total neutralizing antibodies in an isotype- and species-independent manner without requiring a live virus or high biosecurity containment, and thus making the test immediately accessible to the global community [33]. In the present study, VIDAS^®^3 demonstrated the best correlation with the sVNT in detecting IgG antibodies with neutralizing activity against SARS-CoV-2 (Figure 5), which was expected since both assays target the RBD of S1 protein. This suggests that VIDAS®3 could be used for detecting IgG antibodies that correlate with protective immunity. Moreover, concordance assessment among the automated-IgG assays and the sVNT showed a high overall percent agreement, nevertheless, a variation in the positive and negative percent agreements was observed (Table 4). 

In the present study, using sVNT as a reference test, all three automated assays demonstrated a sensitivity above 90%, with the highest overall sensitivity estimated at 96.1% by CL-900i^®^ (Figure 4). Recently, Abbott Architect was reported to have a sensitivity of 80.5%, using microneutralization test (MNT) as a reference method [53]. It is noteworthy to mention that the variation in sensitivity values reported in most studies on the currently available commercial automated analyzers, could be in part due to the variation in the time of sample collection. The sensitivity of serological tests is usually lower at early stages of infection (<7 days), and the performance starts to stabilize ≥ 21 days after symptom onset [8,42]. In correlation to the sVNT, LIAISON^®^XL had a 92.2% positive percent agreement and 100% negative percent agreement (Table 4), this is in concordance with another study reporting positive and negative percent agreements of 94.4% and 97.8%, respectively, for LIAISON^®^XL using MNT as reference method [54].

In the present study, adaptation of lower cut-off values, as determined by the ROC curve analysis (Figure 3), improved the sensitivities of all assays without affecting the specificity. Thus, lower cut-off values may be used to improve the detection of SARS-CoV-2 IgG antibodies by the three assays. Other studies have also suggested using a lower cut-off for LIAISON^®^XL (8.76 AU/mL and 9 AU/mL) [54,55]. The importance of using a cut-off value that provides high sensitivity compared to one that provides low sensitivity, but high specificity is affected by the disease prevalence. For screening purposes, higher thresholds may be desirable, whereas for diagnosis purposes in high-prevalence settings, lower thresholds are preferred. Therefore, it is recommended for each lab to establish its own cut-off values to improve the clinical performance and avoid false-negative results. 

Although serological assays do not replace molecular tests in diagnosing active infection, they serve as an essential tool to accurately estimate the seroprevalence of SARS-CoV-2 infection in the general population and to quantify the level of herd immunity [56]. This could help ease the restrictions on human mobility and interactions without provoking a significant resurgence of transmission and mortality. In addition, serological tests will also help in assessing the potential effectiveness of vaccine trials and antibody-mediated therapies [33,53].

Our study has several limitations. The RT-PCR-confirmed SARS-CoV-2 samples were collected at ≥21 days post symptom onset. Thus, the results obtained for the diagnostic efficiency could have been different if samples at different time points (<21 days) were available. In addition, technical problems such as insufficient sample volume may have affected the results. However, since all samples were drawn in duplicate, we were able to continue the, notably by using multiple aliquotes that were kept in −80 °C for later use.

## 5. Conclusions

In conclusion, the three evaluated automated assays: CL-900i^®^ SARS-CoV-2 IgG (Mindray, China); VIDAS^®^3 SARS-CoV-2 IgG (bioMérieux, France); and LIAISON^®^XL SARS-CoV-2 IgG (Diasorin, Italy), demonstrated high overall sensitivity and specificity for the detection of IgG antibodies against SARS-CoV-2. Among the three automated assays, CL-900i^®^ demonstrated the best diagnostic performance. In addition, VIDAS^®^3 correlated best with the neutralization test, and thus could serve as a tool for detecting protective IgG threshold, particularly in vaccinated population.

## Figures and Tables

**Table 1:** Characteristics of the automated analyzers used for SARS-CoV-2 IgG antibodies detection.

Automated Analyzer	Detection Method	Targeted Antigen(s) ^a^	Sample Volume	Result Interpretation	Reported Sensitivity	Reported Specificity	Reference
VIDAS^®^3	ELFA	S1 RBD	100 μL (including the dead volume)	<1 AU/mL: Negative ≥1 AU/mL: Positive	100% (≥15 days)	98.5%	[29,30]
CL-900i^®^	CLIA	S and N proteins	10 μL (this volume does not include the dead volume)	<10 AU/mL: Negative ≥10 AU/mL: Positive	100% (≥15 days)	94.9%	[27,28]
LIAISON^®^XL	CLIA	S1/S2	170 μL of specimen (20 μL specimen +150 μL dead volume)	<12 AU/mL: Negative 12–15 AU/mL: Borderline >15 AU/mL: Positive	97.5% (≥15 days)	98.2%	[30,31]

ELFA, Enzyme Linked Fluorescent Assay; CLIA, chemiluminescence immunoassay; RBD, receptor-binding domain. ^a^ S1 and S2 are subunits of the spike protein; the RBD is a domain within the S1 subunit; N is the nucleocapsid protein.

**Table 2:** Diagnostic assessment of the three automated analyzers for SARS-CoV-2 IgG detection using RT-PCR as a reference test for the (**A**) overall sample, (**B**) symptomatic patient group, and (**C**) asymptomatic patient group.

		RT-PCR			Overall Percent Agreement	Sensitivity	Specificity	Cohen’s Kappa Statistic
		Positive	Negative	Total	% (95% CI)	% (95% CI)	% (95% CI)	k (95% CI)
(**A**)								
	Negative	11	121	132				
	Total	111	127	238				
VIDAS^®^3 assay	Positive	98	2	100	93.7 (89.9–96.2)	88.3 (81.0–93.0)	98.4 (94.5–99.6)	0.87 (0.83–0.92)
	Negative	13	125	138				
	Total	111	127	238				
CL-900i^®^ assay	Positive	100	6	106	92.9 (88.9–95.5)	90.1 (83.1–94.4)	95.3 (90.1–97.8)	0.86 (0.81–0.90)
	Negative	11	121	132				
	Total	111	127	238				
LIAISON^®^XL assay	Positive	95	0	95	93.3 (89.4–95.8)	85.6 (77.9–90.9)	100 (97.1–100)	0.86 (0.82–0.91)
	Negative	16	127	143				
	Total	111	127	238				
(**B**)								
VIDAS^®^3 assay	Positive	54	2	56	97.8 (94.5–99.1)	96.4 (87.9–99.0)	98.4 (94.5–99.6)	0.95 (0.92–0.98)
	Negative	2	125	127				
	Total	56	127	183				
CL-900i^®^ assay	Positive	55	6	61	96.2 (92.3–98.1)	98.2 (90.6–99.7)	95.3 (90.1–97.8)	0.91 (0.87–0.95)
	Negative	1	121	122				
	Total	56	127	183				
LIAISON^®^XL assay	Positive	51	0	51	97.3 (93.8–98.8)	91.1 (80.7–96.1)	100 (97.1–100)	0.93 (0.90–0.97)
	Negative	5	127	132				
	Total	56	127	183				
(**C**)								
	Negative	10	121	131				
	Total	51	127	178				
VIDAS^®^3 assay	Positive	40	2	42	92.7 (89.8–96.2)	78.4 (64.7–88.7)	98.4 (94.5–99.6)	0.81 (0.75–0.87)
	Negative	11	125	136				
	Total	56	127	178				
CL-900i^®^ assay	Positive	41	6	47	91.0 (85.9–94.4)	80.4 (67.5–89.0)	95.3 (90.1–97.8)	0.77 (0.71–0.84)
	Negative	10	121	131				
	Total	51	127	178				
LIAISON^®^XL assay	Positive	40	0	40	93.8 (89.3–96.5)	78.4 (65.4–87.5)	100 (97.1–100)	0.84 (0.78–0.90)
	Negative	11	121	131				
	Total	51	127	178				

**Table 3:** The specificity of each automated analyzer according to the negative control subgroups (*n* = 127).

Subgroup with IgG/IgM Antibodies Against:	No. of Samples	VIDAS^®^3	CL-900i^®^	LIAISON^®^XL
		% (95% CI)	% (95% CI)	% (95% CI)
Other human CoVs (SARS-CoV, MERS-CoV, HCoV-229E, NL63, OC43, and HKU1)	18	18/18; 100 (82.4–100)	12/18; 66.7 (43.8–83.7)	18/18; 100 (82.4–100)
Non-CoV respiratory viruses (Influenza A and RSV)	38	37/38; 97.4 (86.5–99.5)	38/38; 100 (90.8–100)	38/38; 100 (90.8–100)
Non-respiratory viruses (HEV, HGV, HCV, HBV, DENV, WNV, CHIKV, B19, HSV-1, HSV-2, EBV, HHV-6, and HHV-8)	65	65/65; 100 (94.4–100)	65/65; 100 (94.4–100)	65/65; 100 (94.4–100)
Antinuclear antibodies (ANAs)	6	5/6; 83.3 (43.7–97.0)	6/6; 100 (61.0–100)	6/6; 100 (61.0–100)
Overall specificity	127	125/127; 98.4 (94.5–99.6)	121/127; 95.3 (90.1–97.8)	127/127; 100 (97.1–100)

**Table 4:** Concordance assessment between the sVNT, VIDAS^®^3, CL-900i^®^, and LIAISON^®^XL tests.

Test	Compared to	Overall Percent Agreement	Positive Percent Agreement	Negative Percent Agreement	Cohen’s Kappa Statistic
		% (95% CI)	% (95% CI)	% (95% CI)	k (95% CI)
sVNT	VIDAS^®^3	106/111; 95.5 (89.9–98.1)	98/103; 95.1 (89.1–97.9)	8/8; 100 (67.6–100)	0.74 (0.65–0.83)
	CL-900i^®^	106/111; 95.5 (89.9–98.1)	99/103; 96.1 (90.4–98.5)	7/8; 87.5 (52.9–97.8)	0.71 (0.62–0.81)
	LIAISON^®^XL	103/111; 92.8 (86.4–96.3)	95/103; 92.2 (85.4–96.0)	8/8; 100 (67.6–100)	0.63 (0.52–0.74)
VIDAS^®^3	sVNT	106/111; 95.5 (89.9–98.1)	98/98; 100 (92.3–100)	8/13; 61.5 (35.5–82.3)	0.74 (0.65–0.83)
	CL-900i^®^	105/111; 94.6 (88.7–97.5)	96/98; 98.0 (92.5–99.4)	9/13; 69.2 (42.4–87.3)	0.72 (0.62–0.82)
	LIAISON^®^XL	106/111; 95.5 (89.9–98.1)	94/98; 95.9 (90.0–98.4)	12/13; 92.3 (66.7–98.6)	0.80 (0.72–0.88)
CL-900i^®^	sVNT	106/111; 95.5 (89.9–98.1)	99/100; 99.0 (94.6–99.8)	7/11; 63.6 (35.4–84.8)	0.71 (0.62–0.81)
	VIDAS^®^3	105/111; 94.6 (88.7–97.5)	96/100; 96.0 (90.2–98.4)	9/11; 81.8 (52.3–94.9)	0.72 (0.62–0.82)
	LIAISON^®^XL	104/111; 93.7 (87.6–96.9)	94/100; 94.0 (87.5–97.2)	10/11; 90.9 (62.3–98.4)	0.71 (0.61–0.80)
LIAISON^®^XL	sVNT	103/111; 92.8 (86.4–96.3)	95/95; 100 (96.1–100)	8/16; 50.0 (28.0–72.0)	0.63 (0.52–0.74)
	CL-900i^®^	104/111; 93.7 (87.6–96.9)	94/95; 98.9 (94.3–99.8)	10/16; 62.5 (38.6–81.5)	0.71 (0.61–0.80)
	VIDAS^®^3	106/111 95.5 (89.9–98.1)	94/95; 98.9 (94.3–99.8)	12/16; 75.0 (50.5–89.8)	0.80 (0.72–0.88)

## Data Availability

Not applicable.
